# Mechanisms associated with the synergistic induction of resistance to tobacco black shank in tobacco by arbuscular mycorrhizal fungi and β-aminobutyric acid

**DOI:** 10.3389/fpls.2023.1195932

**Published:** 2023-06-26

**Authors:** Jia Li, Bo Cai, Sheng Chang, Ying Yang, Shuhui Zi, Tao Liu

**Affiliations:** ^1^ College of Agronomy and Biotechnology, Yunnan Agricultural University, Kunming, China; ^2^ National & Local Joint Engineering Research Center on Germplasm Innovation & Utilization of Chinese Medicinal Materials in Southwestern China, Kunming, China; ^3^ Key Laboratory of Medicinal Plant Biology, Yunnan Agricultural University, Kunming, China; ^4^ Technical Center of Yunnan Zhongyan Industry Co., Ltd, Kunming, China

**Keywords:** arbuscular mycorrhizal fungi, β-aminobutyric acid, *Phytophthora nicotianae*, plant disease resistance, tobacco blank shank

## Abstract

Tobacco black shank (TBS), caused by *Phytophthora nicotianae*, is one of the most harmful diseases of tobacco. There are many studies have examined the mechanism underlying the induction of disease resistance by arbuscular mycorrhizal fungi (AMF) and β-aminobutyric acid (BABA) alone, but the synergistic effects of AMF and BABA on disease resistance have not yet been studied. This study examined the synergistic effects of BABA application and AMF inoculation on the immune response to TBS in tobacco. The results showed that spraying BABA on leaves could increase the colonization rate of AMF, the disease index of tobacco infected by *P.nicotianae* treated with AMF and BABA was lower than that of *P.nicotianae* alone. The control effect of AMF and BABA on tobacco infected by *P.nicotianae* was higher than that of AMF or BABA and *P.nicotianae* alone. Joint application of AMF and BABA significantly increased the content of N, P, and K in the leaves and roots, in the joint AMF and BABA treatment than in the sole *P. nicotianae* treatment. The dry weight of plants treated with AMF and BABA was 22.3% higher than that treated with *P.nicotianae* alone. In comparison to *P. nicotianae* alone, the combination treatment with AMF and BABA had increased Pn, Gs, Tr, and root activity, while *P. nicotianae* alone had reduced Ci, H_2_O_2_ content, and MDA levels. SOD, POD, CAT, APX, and *Ph* activity and expression levels were increased under the combined treatment of AMF and BABA than in *P.nicotianae* alone. In comparison to the treatment of *P.nicotianae* alone, the combined use of AMF and BABA increased the accumulation of GSH, proline, total phenols, and flavonoids. Therefore, the joint application of AMF and BABA can enhance the TBS resistance of tobacco plants to a greater degree than the application of either AMF or BABA alone. In summary, the application of defense-related amino acids, combined with inoculation with AMF, significantly promoted immune responses in tobacco. Our findings provide new insights that will aid the development and use of green disease control agents.

## 1 Introduction

Plants are exposed to various environmental threats throughout their life cycle, such as biotic stresses (Kim et al., 2022). Plant pathogenic fungi are one of the major biotic stresses that negatively affect plant productivity worldwide. To defend themselves against these pathogenic fungi, plants have evolved disease-fighting mechanisms similar to the innate immune mechanisms of animals (Yu et al., 2022). Tobacco blank shank (TBS), a soil-borne fungal disease caused by *Phytophthora nicotianae Breda* has caused severe economic losses in tobacco production (Vontimitta and Lewis, 2010). Traditional control strategies, such as the spraying of chemical pesticides, the selection of disease-resistant varieties, and other measures, have various shortcomings (Damalas and Eleftherohorinos, 2011). Because of the negative environmental effects of pesticides and their implications for ensuring agricultural productivity and environmental safety, development of efficient and environmentally friendly TBS control strategies is key for improving the yield and quality of tobacco. Plant-induced resistance is a safe and effective approach for the control of TBS. Using the plant immune system to control diseases can reduce contamination of the environment and agricultural products by pesticides. The use of safe and environmentally friendly amino acid compounds and biocontrol fungi to enhance resistance to diseases in plants has been explored (Justyna and Ewa, 2013; Wang et al., 2022). However, few studies have evaluated the control efficacy of applying more than one of these agents.

As shown in many studies, induction of resistance in the belowground parts of plants is usually achieved *via* beneficial microorganisms such as plant growth-promoting rhizobacteria (Jiao et al., 2021) and arbuscular mycorrhizal fungi (AMF). AMF are beneficial fungi that form mycorrhizae during symbiosis with the root system of vascular plants (Shi et al., 2017). AMF provide nutrients to plants (Wang et al., 2019), compete with pathogens (Li et al., 2019), alter plant root morphology (Hou et al., 2018), alter plant rhizosphere microflora (Bagy et al., 2019), and induce changes in the activity of secondary metabolites and enzymes in plants (Jiang et al., 2015; Lian et al., 2019), which enhances plant disease resistance. Melatonin improves the resistance of cucumber plants to *Fusarium* wilt by increasing plant dry weight, leaf gas exchange parameters, and the activity of antioxidant enzymes in leaves infected by *Fusarium oxysporum.* However, the relationship between changes in the content of nitrogen (N), phosphorus (P), and potassium (K) in the roots and improvements in disease resistance has not yet been clarified (Ahammed et al., 2020). Tomato plants inoculated with mycorrhizae show increased antioxidant enzyme activity, which affects the elimination of reactive oxygen species such as hydrogen peroxide (H_2_O_2_) and reduces the effects of *Fusarium* wilt on plants (Hashem et al., 2018). However, changes in H_2_O_2_ and malondialdehyde (MDA) levels were not measured. Most previous studies have examined the effects of applying AMF and other microorganisms on the disease resistance of host plants (Liu et al., 2017), however, the efficacy of applying other inducers of resistance along with AMF for enhancing the stress resistance of hosts has not yet been evaluated (Ahammed et al., 2020; Lin et al., 2020). Although there are many studies have examined the symbiosis between AMF and tobacco (Guo et al., 2015), how AMF mediates increases in the resistance of tobacco to TBS has not been evaluated, furthermore, the mechanisms underlying the increase in resistance induced by AMF and DL-β-aminobutyric acid (BABA) have not been elucidated.

Disease resistance in plants is typically induced *via* measures such as spraying jasmonic acid (Lin et al., 2020), melatonin (Tiwari et al., 2022), and amino acid compounds. BABA induces disease resistance in plants by acting as an ‘elicitor’. It mainly stimulates host resistance to pathogens by inducing plants to produce physiological barriers (Baccelli and Mauch-Mani, 2016), producing hypersensitive response (Pathogenesis-related proteins specifically recognized the effectors of pathogens to stimulate the effector - triggered immunity immune response of plants. Effector - triggered immunity can accelerate and amplify the pathogen-associated molecular patterns-triggered immunity responsed, resulting in disease resistance in plants. At the same time, local programmed cell death will occur near the infection point to inhibit the spread of pathogens.) and reactive oxygen species (Mahmud et al., 2020), accumulating pathogenesis-related proteins (Li et al., 2022), inducing plants to produce phytoncides (Liao et al., 2018), and initiating specific signaling pathways (Jakab et al., 2001; Hamiduzzaman et al., 2005). BABA induces disease resistance in *Lycium barbarum L* by increasing the H_2_O_2_ content, reducing the MDA content, increasing superoxide dismutase (SOD) and peroxidase (POD) activities, and increasing total phenol and flavonoid levels, however, the expression levels of enzyme-encoding genes and disease resistance genes in *Lycium barbarum L* have not been quantified (Zhang et al., 2022). BABA treatment can protect rapeseed from salt stress by enhancing the antioxidant defense system, inducing the up-regulation of the expression of genes encoding antioxidant enzymes, such as superoxide dismutase (SOD), catalase (CAT), and ascorbate peroxidase (APX), which might play a role in removing free radicals, enhancing plant defense, and reducing oxidative stress (Mahmud et al., 2020). Spraying application of BABA on tobacco leaves induces an allergic reaction in the leaves, which forms a dead spot that inhibits the invasion of the virus. It also promotes a large increase in reactive oxygen species in tobacco cells; an increase in the activity of peroxidase (POD), which scavenges H_2_O_2_; and a decrease in the activity of CAT, which is the main enzyme scavenging H_2_O_2_, all of which contribute to increases in the resistance of tobacco to tobacco mosaic virus (He et al., 2010). Although much research on the role of BABA in plants has been reported, the mechanisms involved in inducing resistance to TBS in tobacco remain unclear.

In this study, we determined whether inoculation with AMF and BABA synergistically induces the systemic resistance of tobacco to the fungal pathogen *P. nicotianae*. We evaluated physiological and biochemical indicators, antioxidant enzyme genes, and genes related to TBS resistance to investigate the mechanisms involved in the synergistic induction of resistance to TBS in tobacco by AMF and BABA. Additional studies of the aboveground application of defensive amino acid compounds and inoculation with AMF will aid the development of green disease control agents.

## 2 Materials and methods

### 2.1 Materials and experimental design

Plants were grown under normal temperature conditions in the plastic house of Yunnan Agricultural University from May 15 to October 30. The average annual temperature was 15.1°C, the average temperature of the hottest month (July) was 19.7°C, the average temperature of the coldest month (January) was 7.5°C, the annual temperature difference was 12.0-13.0°C, the annual precipitation was approximately 1 031 mm, and the relative humidity was 74%.

The tobacco variety Honghuadajinyuan (HD) was used because it is susceptible TBS. The *Phytophthora nicotianae* strain used was isolated by our laboratory and sequenced by PCR and then compared with sequences in the National Center for Biotechnology Information databases. The mycelium was sealed in glycerol and stored in a refrigerator at-80°C. It was recultured on potato dextrose agar (PDA: 200g of potato, 20g of glucose (Kunming Aoxue Co., Ltd., Yunnan, China) and 15g of agar (Kunming Aoxue Co., Ltd., Yunnan, China), and 1000ml of water) in a constant temperature incubator at 30°C for 15 days. AMF spores from *Paraglomus occultum* (C. Walker) J.B. Morton & D. Redecker (Beijing Institute of Plant Nutrition and Resource Environment, Beijing, China) and BABA were purchased from Sigma, USA, and used to induce disease resistance in tobacco plants.

A pot experiment was conducted was designed with eight treatments: (1) no spraying of BABA, no inoculation with AMF, and no infection with *P. nicotianae* (CK); (2) only inoculation with AMF (AMF); (3) only spraying of BABA (BABA); (4) spraying of BABA and inoculation with AMF (AMF+BABA); (5) infection with *P. nicotianae*; (6) inoculation with AMF and infection with *P. nicotianae* (AMF + *P. nicotianae*); (7) spraying with BABA and infection with *P. nicotianae* (BABA + *P. nicotianae*); and (8) spraying with BABA, inoculation with AMF and infection with *P. nicotianae* (AMF + BABA + *P.nicotianae*). The experimental was conducted in completely randomized design with 15 replications of per treatment and a total of 120 pots.

### 2.2 AMF inoculation, BABA spraying, and *P. nicotianae* infection

Tobacco plants were transplanted when the seedlings reached the three-leaf stage; there was one plant in each pot, and the size of each plant was the same. The AMF inoculum was added near the roots of seedlings of AMFtreated plants. The AMF inoculum contained spores, soil, and corn root segments, andapproximately 680 spores per gram were added to each pot in a total of 10 g. Plants not treated with AMF treatment were inoculated with the same amount of AMF inoculum sterilized by high pressure, and colonization by AMF was observed 42 days after inoculation. Water and Hoagland nutrient solution was applied every 2 days after tobacco plants were transplanted until water overflowed the pot. Starting on the fifth day, 35 ml of BABA (2.5 mM) was sprayed on the plants every 5 days. After 30 days, 35 ml of BABA (5 mM) was sprayed on the plants, and sterile water was sprayed on plants as a control treatment. After 30 days of BABA treatment, the stems of tobacco plants in the appropriate treatments were infected with *P. nicotianae*. After 30 days of *P. nicotianae* infection, the plants showing clear signs of TBS infection were sampled (Ren et al., 2022). Three and four leaves of the plants (from top to bottom) were sampled, and the samples were immediately stored in liquid nitrogen for the subsequent determination of various indexes.

### 2.3 Determination of the arbuscular mycorrhizal fungal colonization rate, disease index, and control effectiveness

Determination of AMF reference trypan blue staining: (1) Fixation: Freshly harvested roots were chopped into pieces approximately 1 cm long and stored at 4 °C in formaldehyde-alcohol-acetic acid (FAA) fixative solution. (2) Transparency: The root segments were washed with distilled water, immersed in 10% KOH solution, and heated in a 90°C water bath for 60 min until they became a slightly white and transparent color. (3) Acidification: The residual KOH on the roots was cleaned with distilled water, immersed in 2% HCL solution, acidified for 10 min, and then cleaned with distilled water. (4) Staining: The washed root segments were placed in 0.05% trypan blue staining solution and heated in a water bath at 90°C for 30 min. After cooling, they were placed in lactic acid glycerol solution for decolorization for more than 60 min (Yang et al., 2010). Twenty sections of roots were measured per treatment, and observations of roots that had been colonized with AMF were made. Plants were then placed in the following categories depending on the extent of AMF colonization in each root segment: 0, <1%, <10%, <50%, >50%, or >90%. Calculations were carried out using the following formula: AMF colonization rate =∑M%•n/N, where M% represents the colonization rate of individual root segments, n represents the number of root segments with the same colonization rate, and N represents the total number of root segments per treatment (Biermann and Linderman, 1983).

Disease indices were determined based on grades of TBS infection severity and the vascular browning of tobacco plants. The following scale was used to classify the severity of TBS: Grade 0, the whole plant was free of disease; Grade 1, the stem spot did not exceed one-third of the stem circumference, or less than one-third of the leaves showed blight; Grade 3, stem spot encircled one-third to half of the stem circumference, one-third to half of the leaves showed slight blight, or a few lower leaves showed blight; and Grade 9, the diseased plant was dead. TBS disease severity was assessed by the ratio of stem lesions to the stem circumference or the proportion of whole plant leaves that died (Ren et al., 2022). The disease index (DI) was calculated using the following formula: DI= [∑(grading score × number of plants graded)/(total number of plants × highest grading score) × 100]. The control effect was estimated from the DI as follows: relative control effect (%) = [(control DI -treatment DI)/control DI] × 100.

### 2.4 Gas exchange parameters

Fifteen days after inoculation with the TBS pathogen *P. nicotianae*, air exchange was measured for the second fully expanded leaf (from top to bottom) of five of the tobacco plants from 8:00 am to 11:00 am. Data on gas exchange parameters such as the net photosynthetic rate (Pn), intercellular CO_2_ concentration (Ci), stomatal conductance (Gs), and transpiration rate (Tr) were generated using a portable photosynthetic system with an open infrared gas analyzer (LI-6400; LICOR, Lincoln, NE, United States) with a light-controlled, temperature-controlled system; one sample was taken from each leaf. In the gas exchange experiments, the air temperature was maintained at 25 °C, the relative humidity was maintained at 80–90%, the flow rate was 500 µmol/s CO_2_, and the light intensity was 1,000 µmol/(m^2^ s).

### 2.5 Root activity, H_2_O_2_ levels, and MDA levels

The root activity, H_2_O_2_ content, and MDA content were measured using a root activity test kit, H_2_O_2_ content test kit (Suzhou Grace Biotechnology Co., Ltd., Suzhou, China), and MDA content test kit (Suzhou Grace Biotechnology Co., Ltd., Suzhou, China), respectively, according to the manufacturer’s protocols.

### 2.6 Antioxidant enzyme activity assay

The activities of the main antioxidant enzymes, including superoxide dismutase (SOD), peroxidase (POD), catalase (CAT) and ascorbate peroxidase (APX) were measured. SOD assay kits (Suzhou Grace Biotechnology Co., Ltd., Suzhou, China), POD assay kits, CAT assay kits, and APX assay kits (Beijing Box Biotechnology Co., Ltd., Beijing, China) were used to determine the activities of SOD, POD, CAT, and APX, respectively, per the manufacturers’ protocols.

### 2.7 Expression levels of defense-related genes

The expression levels of antioxidant enzyme genes, such as *APX*, *SOD*, *CAT*, and *POD* and the defense-related gene *Ph* (Tong et al., 2014) involved in resistance to TBS, were determined by real-time quantitative PCR (qRT-PCR). Total RNA was extracted from approximately 100-mg leaf samples using a total RNA extraction kit (Beijing Kangrun Chengye Biotechnology Co., Ltd., Beijing, China) per the manufacturer’s instructions; three biological replicates were performed for each sample. One unit of total RNA was reverse-transcribed using the GenStar kit (Beijing Kangrun Chengye Biotechnology Co., Ltd., Beijing, China) to generate cDNA. The reactions were conducted by mixing 1 μg of RNA template, 1.6 μl of 5 × gDNA Remover Reaction Mix, 0.5 μl of gDNA Remover, and 4.9 μl of ddH_2_O_2_ in the order they are listed, followed by centrifugation. The reactions were run at 37 °C for 5 min. In the above reaction tube, 1 μl of ddH_2_O, 10 μl of 2 × RT Reaction Mix (with primer), and 1 μl of StarScript II RT Mix were added in the order they are listed. They were then blended gently, centrifuged briefly, and incubated at 42 °C for 20 min. The StarScript II RT Mix was inactivated by heating at 85 °C for 5 min. The cDNA obtained after the reaction was stored at -20 °C for subsequent experiments. First-strand cDNA was used for reverse transcription and used as a template for qRT-PCR reactions. qRT-PCR was performed using an RT-PCR detection system. The 20 μL reaction system contained the following: 1 μL of cDNA, 10 μL of SYBR Green PCR Master Mix, 1 μL each of forward primer and reverse primer, and 7 μL of ddH_2_O. We used SYBR Green PCR Master Mix for qRT-PCR reactions. The thermal cycling conditions for the qRT-PCR reactions were as follows: 40 cycles of 95 °C for 30 s, 60°C for 25 s, and 72 °C for 30 s. Fluorescence data were collected at the end of the annealing stage in each cycle. The 18S gene was used as an internal standard after conducting preliminary tests in our laboratory. After the forward primer and reverse primer of the 18S gene were synthesized, no primer dimers were observed, which indicated that the 18S gene was expressed. Relative gene expression levels were calculated using the 2^-ΔΔC (T)^ method described in a previous study (Livak and Schmittgen, 2001), and three sets of reactions with the specific primer sequences in **Table 1** were conducted to amplify the genes. Primer synthesis was completed by Beijing Qingke Biotechnology Co., Ltd. (Kunming) (**Table 1**).

**Table 1:** Specific primers for fluorescent quantitative PCR. *SOD*, *POD*, *CAT*, *APX*, *18SRNA* and *Ph*’s forward primers and reverse primers sequence.

Primer names	Forward primer (5’-3’)	Reverse primer (5’-3’)
SOD	CCACCAGAAGCATCATCAGA	TTCCCAGAAGAGGAACCAAA
POD	CACCCCAACTGATGTTGTTG	TTGGCCCTAATTTCACCTTG
CAT	TCATGCTAGAGGTGCCAGTG	CTGTCGGGAGACAAACATCA
APX	CTGAGCAAGGACATGGAGCA	CCACCACTCCCAACTCTTCC
18sRNA	TTCCGTTAACGAACGAGACC	TGTCGGCCAAGGCTATAAAC
Ph	ATCGCTTTGTACTGGCTGCT	CTCTTCAAAACCCTTGCCGC

### 2.8 The contents of N, P, K, antioxidants and secondary metabolites in the roots and leaves were determined

The effective N level was determined using the Kjeldahl method, the effective P level was determined using the Olson method, and the effective K level was determined using a flame spectrophotometer. A GSH assay kit, proline assay kit, total phenol assay kit, and flavonoid assay kit were used to determine the content of reduced GSH, proline, total phenols, and flavonoids (Suzhou Grace Biotechnology Co., Ltd., Suzhou, China), respectively, per the manufacturer’s protocol.

### 2.9 Statistical analysis

Data were input into Microsoft Excel 2016 software, and SPSS 26 software was used to analyze the data. SPSS Statistics 26 software was used to conduct normality tests and analysis of variance. Origin 2019b 64Bit software was used to make figures. Our data were tested for normal distribution, so we used Tukey’s multiple comparison test to assess the significance of differences between treatments at the same time point (P < 0.05). Excel 2016 software was used to input data and SPSS 26 software was used to analyze the data.

## 3 Results

### 3.1 Effect of BABA on the AMF colonization rate and TBS resistance of tobacco

**Figure 1:**
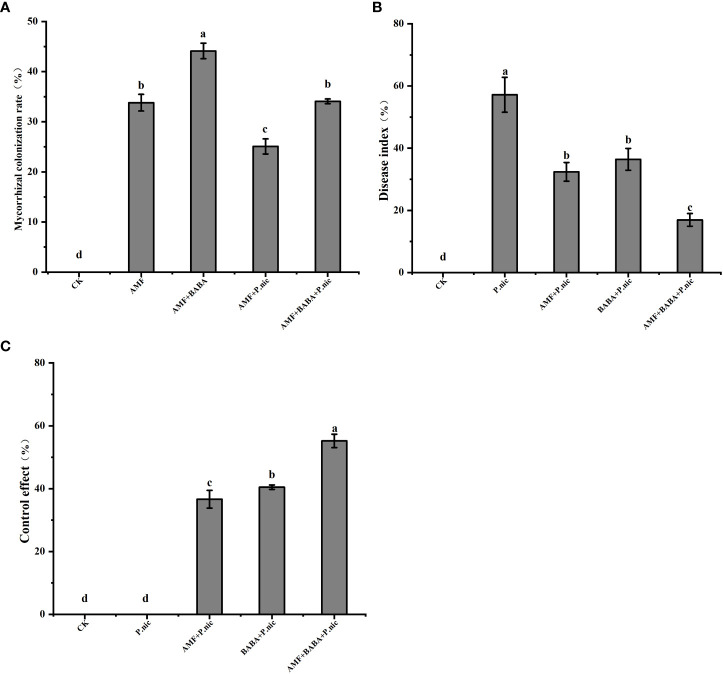
Effects of BABA on colonization of tobacco by AMF in the presence of *P.nicotianae*. **(A)** The colonization rate of AMF under spraying BABA and infection with P.nicotianae (%), **(B)** Disease index (%) and **(C)** Control effect (%). The bar chart represents the average value, and the error bar represents the standard deviation (n = 5). Different lowercase letters on the bar chart indicated significant differences between treatments based on the Tukey test (P < 0.05).

After 35 days of AMF colonization, the colonization rate of AMF when applied alone was 33.8%. Exogenous spraying of BABA significantly increased the colonization rate of AMF by 30.5%, and *P. nicotianae* infection significantly decreased the colonization rate of AMF by 25.8%. AMF colonization was 35.8% higher in the AMF + BABA + *P. nicotianae* treatment than in the AMF + *P. nicotianae* treatment (**Figure 1A**). Both AMF and BABA application reduced DI, and the DI of tobacco plants was 43.3% and 36.3% lower in the AMF + *P. nicotianae* and BABA + *P. nicotianae* treatments than in the sole *P. nicotianae* treatment. The disease index of tobacco plants was 70.3% lower in the AMF + BABA + *P. nicotianae* treatment than in the sole *P. nicotianae* treatment (**Figure 1B**). Disease control effect was 50.6% and 36.3% higher in the AMF + BABA + *P. nicotianae* treatment than in the sole AMF and BABA treatments. Both AMF and BABA induced resistance to TBS, but TBS resistance was highest when AMF and BABA were jointly applied (**Figure 1C**).

### 3.2 Effect of AMF and BABA on the N, P, and K levels of plants and the dry weight of tobacco plants infected with *P. nicotianae*

The N, P, and K levels in the root system were 15.5%, 43%, and 39.4% lower, respectively, in tobacco plants infected with *P. nicotianae* than in control plants. N, P, and K levels were 16.7%, 125.3%, and 129.3% as well as 38.3%, 184.3%, and 151.4% higher in the root system of tobacco plants in the AMF + *P. nicotianae* and BABA + *P. nicotianae* treatments, respectively, than in the root system of tobacco plants infected with *P. nicotianae*; N, P, and K levels were 49.9%, 148.2%, and 185.4% higher in tobacco roots in the AMF + BABA + *P. nicotianae* treatment than in the root system of tobacco plants in the sole *P. nicotianae* treatment, respectively (**Table 2**).

**Table 2:** Content of total nitrogen, phosphorus and potassium in tobacco roots of each treatment.

Treatment	Total nitrogen (N,g/kg)	Total phosphorus (P,g/kg)	Total potassium (K,g/kg)
CK	17.71 ± 0.83451de	2.61 ± 0.24173c	7.92 ± 0.26502d
AMF	24.64 ± 1.98218b	3.63 ± 0.5021abc	14.05 ± 1.9938b
BABA	18.92 ± 0.84445d	2.99 ± 0.10583bc	10.68 ± 0.50764c
AMF+BABA	29.39 ± 3.04791a	4.78 ± 0.61647a	18.33 ± 0.97991a
P.nic	14.96 ± 0.62746e	1.48 ± 0.28449d	4.80 ± 0.36019e
AMF+P.nic	17.46 ± 0.88425de	3.35 ± 0.61992bc	11.01 ± 0.89612c
BABA+P.nic	20.68 ± 0.93874cd	4.22 ± 1.03558ab	12.07 ± 0.42922bc
AMF+BABA+P.nic	22.43 ± 0.69735bc	3.69 ± 0.13abc	13.71 ± 2.29b

The N, P, and K levels in the leaves were 31.9%, 46.3%, and 21.1% lower in the leaves of tobacco plants infected with *P. nicotianae* than in the leaves of control plants, respectively. The N, P, and K levels in tobacco leaves were 76.9%, 54.6%, and 29.9% as well as 80.6%, 92.6%, and 42.8% higher when AMF or BABA were applied alone than in the sole *P. nicotianae* treatment, respectively. The N, P, and K levels in tobacco leaves were 137.7%, 237.4%, and 66.8% higher when AMF and BABA were jointly applied than in the sole *P. nicotianae* treatment, respectively. This indicates that both AMF and BABA could alleviate infection by *P. nicotianae* and increase N, P, and K levels in tobacco, suggesting that the joint application of AMF and BABA was most effective for enhancing TBS resistance (**Table 3**).

**Table 3:** Content of total nitrogen, phosphorus and potassium in tobacco leaves of each treatment.

Treatment	Total nitrogen (N.g/kg)	Total phosphorus (P,g/kg)	Total potassium (K,g/kg)
CK	13.37 ± 1.11e	2.27 ± 0.36896bc	17.8 ± 0.96907e
AMF	18.46 ± 1.035918cd	2.32 ± 0.4884abc	22.28 ± 1.43628cd
BABA	27.55 ± 1.58513b	2.88 ± 0.27154b	26.10 ± 0.79563b
AMF+BABA	31.58 ± 1.19528a	3.89 ± 0.39357a	36.66 ± 1.09235a
P.nic	9.1 ± 0.7766f	1.22 ± 0.15524cd	14.04 ± 0.875f
AMF+P.nic	16.09 ± 2.16925de	1.88 ± 0.20648cd	18.25 ± 0.2488e
BABA+P.nic	16.43 ± 3.26045de	2.35 ± 0.47149bc	20.05 ± 3.1194de
AMF+BABA+P.nic	21.63 ± 3.03979c	4.11 ± 1.0405a	23.43 ± 2.14617bc

**Figure 2:**
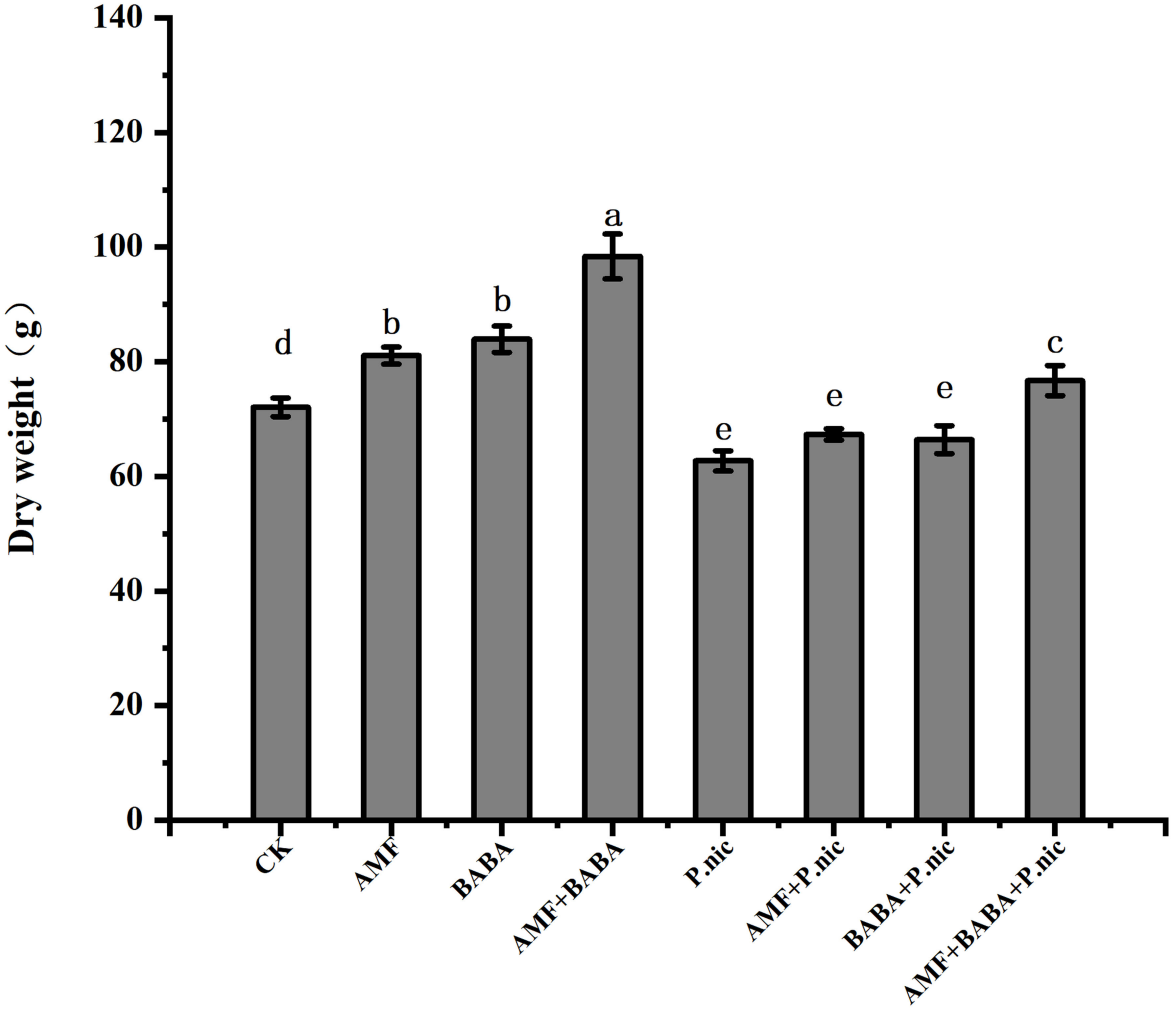
Effect of AMF and BABA on the whole plant dry weight of *P. nicotianae-*infected tobacco. Bars indicate the standard deviation of the mean (n=5). Different lowercase letters on the bars indicate significant differences between treatments based on Tukey’s test (P<0.05). The data shown are the means of five replicates (n=5). Values marked with lowercase letters in the same row differ statistically between treatments (P<0.05).

The dry weight of *P. nicotianae*-infected plants was 13% lower than that of control plants, and the dry weight of plants in the sole AMF and BABA treatments was 7.4% and 5.9% higher than that of plants in the sole *P. nicotianae* treatment, respectively. The dry weight of tobacco plants was 22.3% higher in the joint AMF and BABA treatment than in the sole *P. nicotianae* treatment. Although the dry weight of *P. nicotianae*-infected tobacco plants was increased in the sole AMF and BABA treatments, the dry weight of *P. nicotianae*-infected tobacco plants was increased the most in the joint AMF and BABA treatment (**Figure 2**).

### 3.3 Effect of AMF and BABA on leaf gas exchange parameters in tobacco plants infected with *P. nicotianae*

**Figure 3:**
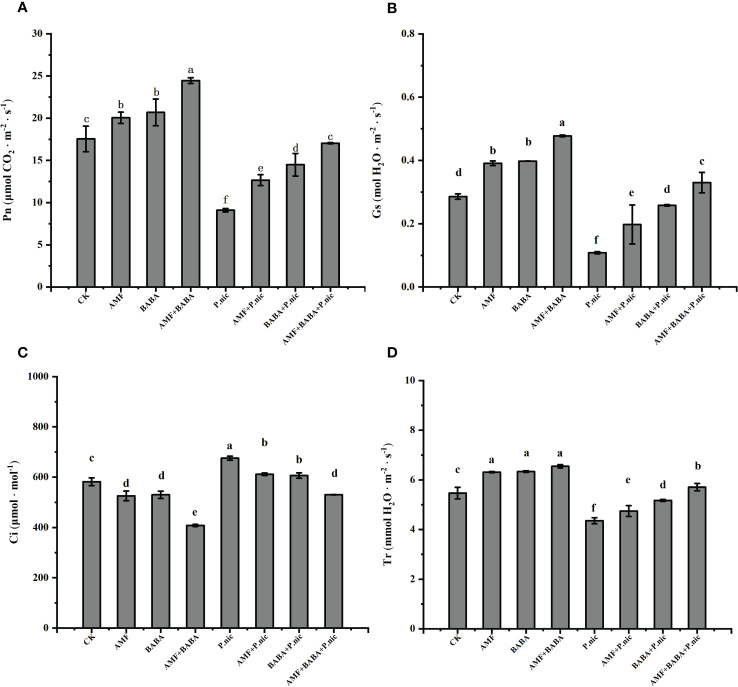
Effect of AMF and BABA on leaf gas exchange parameters in *P. nicotianae*-infected tobacco. Pn **(A)**, Gs **(B)**, Ci **(C)**, and Tr **(D)**. Bars indicate the standard deviation of the mean (n=5). Different letters on the bars indicate significant differences between treatments based on Tukey’s test (P<0.05).

The analysis of gas exchange parameters in leaves showed that compared with the control, *P.nicotianae* significantly reduced Pn, Gs and Tr by 48.1%, 62.2% and 22.3%, respectively, and increased Ci by 16.1%, indicating that the decrease of stomatal conductance after inoculation with *P.nicotianae* may be due to the weakening of CO_2_ assimilation and transpiration. Although neither AMF nor BABA treatment alone affected Pn, the joint application of AMF and BABA significantly increased the CO_2_ assimilation rates in uninfected plants. Compared with the treatment of *P.nicotianae* alone, in the sole AMF or BABA treatment increased the Pn, Gs, and Tr parameters of *P.nicotianae*-infected plants by 39.1%, 82.5%, 8.9% and 59.2%, 138.6%, 18.7%, respectively, and decreased Ci by 9.4% and 10.2%, while the joint application of AMF and BABA increased the Pn, Gs, and Tr parameters of *P.nicotianae*-infected plants by 87.2%, 204.8%, 31.1%, and decreased Ci by 21.5%. This indicates that combined AMF and BABA treatment had an additive effect on leaf gas exchange in tobacco plants after infection with *P. nicotianae* (**Figure 3**).

### 3.4 Effects of AMF and BABA on oxidative stress caused by *P. nicotianae* infection

**Figure 4:**
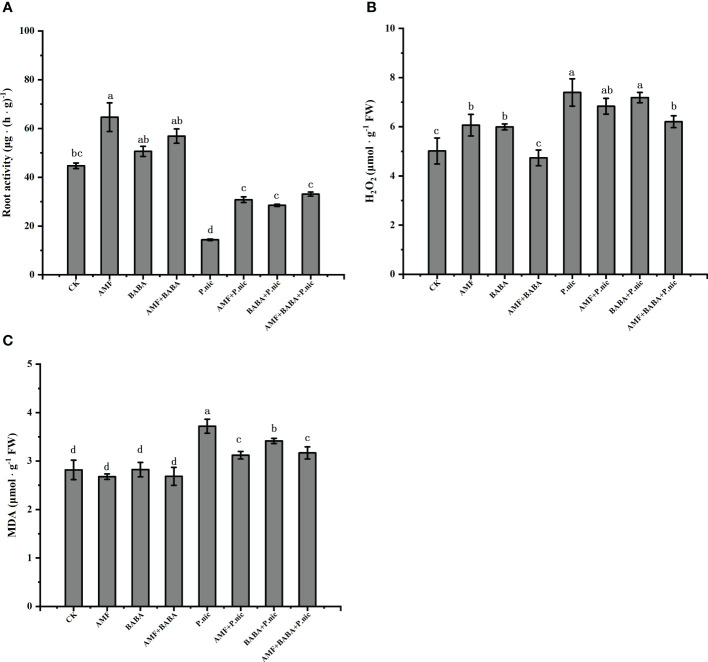
Effects of AMF and BABA on root activity, reactive oxygen species accumulation, and membrane stability in *P. nicotianae* infected tobacco plants. Root activity **(A)**, H_2_O_2_ content **(B)**, and MDA content **(C)**. Bars indicate the standard deviation of the mean (n=5). Different letters on the bars indicate significant differences between treatments based on Tukey’s test (P<0.05).

We investigated the effect of AMF and BABA on the TBS resistance of *P. nicotianae* roots by measuring root activity. Compared with the control, the root activity decreased by 67.9% after *P.nicotianae* infection. In the sole AMF or BABA treatmentincreased the root activity of *P.nicotianae* by 114.3% and 98.3%, respectively, while the combination of AMF and BABA increased the root activity of *P.nicotianae* by 130.1% (**Figure 4A**). This indicated that both AMF and BABA could enhance the resistance of tobacco roots to *P.nicotianae* infection, and the joint application of AMF and BABA was better than single application, but there was no significant difference.

We measured H_2_O_2_ and MDA levels in tobacco leaves to explore the mechanism by which root activity induced TBS resistance in the aboveground parts. Compared with the control, *P.nicotianae* increased H_2_O_2_ and MDA levels by 47.4% and 31.9%, respectively. In the sole AMF or BABA decreased H_2_O_2_ accumulation and MDA content by 7.5%, 2.8% and 16.1%, 8.1%, respectively, the combination of AMF and BABA decreased H2O2 and MDA content by 16.1% and 14.8%, respectively. This indicates that AMF and BABA application had synergistic effects in inducting the TBS resistance of *P. nicotianae*-treated plants. H_2_O_2_ and MDA levels were 16.1% and 14.8% lower in *P. nicotianae*-infected plants in the joint AMF and BABA treatment than in the sole *P. nicotianae* treatment, respectively (**Figures 4B, C**), indicating that both AMF and BABA could alleviate oxidative stress in tobacco by reducing H_2_O_2_ accumulation and the MDA content and that the combined AMF and BABA treatment was more effective than the sole treatment of either one.

**Figure 5:**
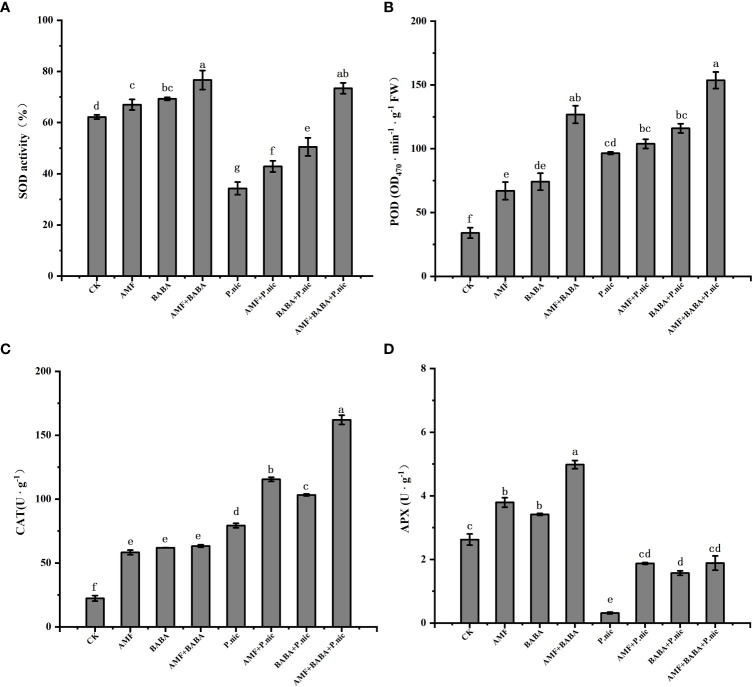
Effect of AMF and BABA on antioxidant enzyme activity in *P. nicotianae*-infected tobacco. SOD activity **(A)**, POD content **(B)**, CAT content **(C)**, and APX content **(D)**. Bars indicate the standard deviation of the mean (n=5). Different letters on the bars indicate significant differences between treatments based on Tukey’s test (P<0.05).

We compared the activities of some key antioxidant enzymes under different treatments (**Figure 5**). The results showed that compared with the control, the activities of SOD and APX in tobacco leaves infected with *P.nicotianae* decreased by 44.9% and 88%, while the activities of POD and CAT increased by 183.8% and 254.5%, respectively. The activities of SOD, POD, CAT and APX in tobacco leaves infected by *P.nicotianae* were increased by 25.1%, 7.6%, 45.6%, 494.3% and 47.3%, 20.2%, 30.2%, 398.1%, respectively, when treated with AMF or BABA alone. The activities of SOD, POD, CAT and APX in tobacco leaves infected by *P.nicotianae* were significantly improved by the joint application of AMF and BABA. The activities of SOD, POD and CAT in the leaves of *P.nicotianae* infected by the joint application of AMF and BABA were increased by 114.1%, 59.2%, 104.3% and 498.1%, respectively, which were 4.5 times, 7.8 times and 2.3 times of those in the leaves of *P.nicotianae* infected by AMF alone, and 2.4 times, 3 times and 3.5 times of those in the leaves of BABA. These findings indicate that the sole AMF and BABA treatments can increase the activities of antioxidant enzymes and that the joint application of AMF and BABA increases the activities of these enzymes to a greater degree than the sole AMF and BABA treatments, suggesting that the joint application of AMF and BABA can help plants resist *P. nicotianae* infection by improving the activities of antioxidant enzymes.

**Figure 6:**
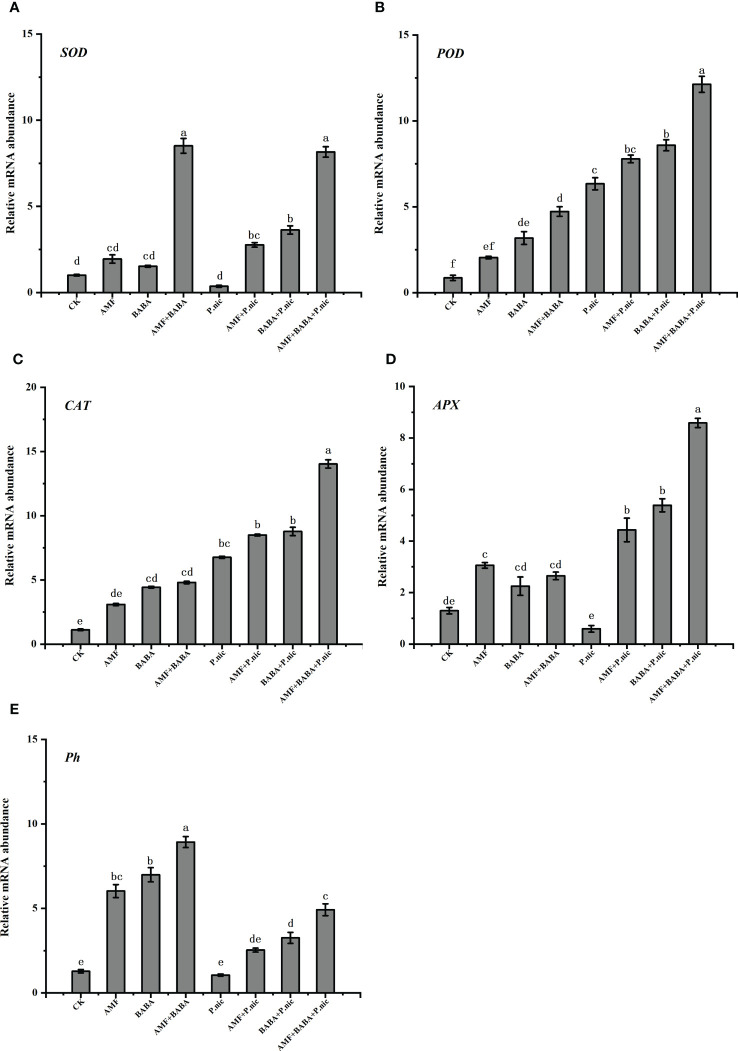
Effect of AMF and BABA on the transcript levels of antioxidant genes in *P. nicotianae*-infected tobacco. *SOD* gene transcript levels **(A)**, *POD* gene transcript levels **(B)**, *CAT* gene transcript levels **(C)**, *APX* gene transcript levels **(D)**, and black shank resistance gene (*Ph*) transcript levels **(E)**. Bars indicate the standard deviation of the mean (n=5). Different letters on the bars indicate significant differences between treatments based on Tukey’s test (P<0.05).

Compared with the control, the expression of *SOD* and *APX* in *P. nicotianae*-infected plants was inhibited, and their transcription levels were down-regulated by 63.8% and 54.4%, the transcription levels of *POD* and CAT were up-regulated by 633.1% and 504.7%, respectively. Compared with the sole *P. nicotianae*-infected, the expression of *SOD*, *POD*, *CAT*, and *APX* were increased by 659.7%, 22.8%, 25.6%, and 649.4% as well as 897.9%, 35.3%, 29.7%, and 897.9% in the leaves of the sole AMF or BABA treatments respectively. The expression of *SOD*, *POD*, *CAT*, and *APX* was 2,140.6%, 91.3%, 107.4%, and 1,352.2% higher in the leaves of *P. nicotianae*-infected plants in the joint AMF and BABA treatment than in the sole *P. nicotianae* treatment, respectively. The expression of *SOD*, *POD*, *CAT*, and *APX* was 3.3, 4, 4, and 2 times higher as well as 2.4, 2.6, 3, and 1.7 times higher in the leaves of *P. nicotianae*-infected plants in the joint BABA and AMF treatment than in the sole AMF and BABA treatments, respectively. Therefore, treatment with AMF and BABA can increase the expression of antioxidant enzymes, and the joint AMF and BABA treatment had a stronger effect on the expression of these genes in *P. nicotianae*-infected plants, indicating that the joint AMF and BABA treatment can increase the resistance of plants to *P. nicotianae* infection by regulating the expression of antioxidant enzyme genes (**Figure 6**). To determine the expression of TBS-related genes (e.g., *Ph*) involved in AMF- and BABA-induced resistance, we used real-time quantitative PCR to analyze the expression of the *Ph* gene in the leaves of tobacco plants after they were infected with *P. nicotianae*. The expression of the *Ph* gene was 140% and 208.2% higher in *P. nicotianae*-infected plants in the sole AMF and BABA treatments, respectively, than in the sole *P. nicotianae* treatment, and the expression of the *Ph* gene was 365.8% higher in the leaves *P. nicotianae*-infected plants in the joint AMF and BABA treatment than in the sole *P. nicotianae* treatment. The expression of the *Ph* gene was 2.6 and 1.5 times higher in the leaves of *P. nicotianae*-infected plants in the sole AMF and BABA treatments, respectively, than in the sole *P. nicotianae* treatment, which indicates that AMF and BABA can both increase the resistance of plants to *P. nicotianae* infection by up-regulating the expression of anti-TBS genes; however, the up-regulation of anti-TBS genes was highest in the joint AMF and BABA application (**Figure 6E**).

### 3.5 Effects of AMF and BABA on the accumulation of antioxidants and secondary metabolites in *P. nicotianae*-infected tobacco plants

**Figure 7:**
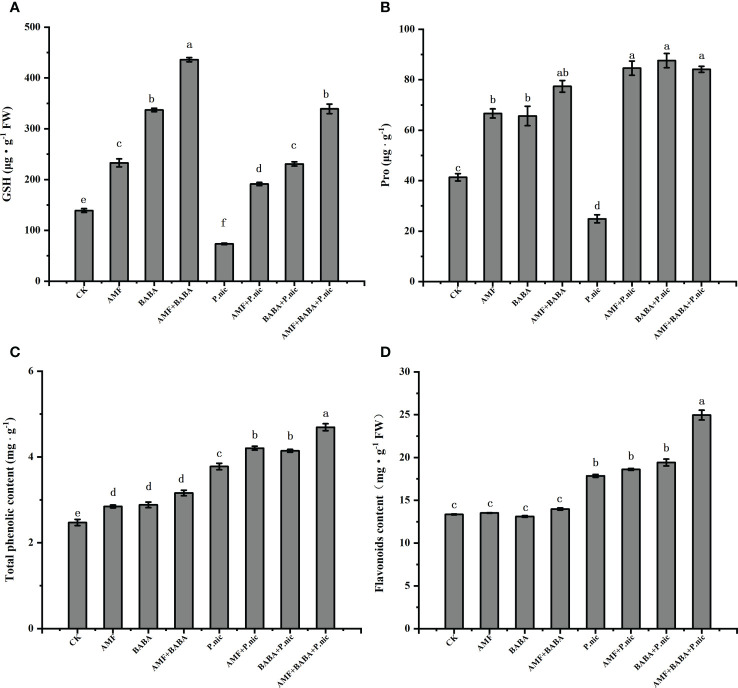
Effect of AMF and BABA on the levels of nonenzymatic antioxidants and secondary metabolites in leaves of *P. nicotianae* infected tobacco. Levels of reduced GSH **(A)**, proline **(B)**, total phenolics **(C)**, and flavonoids **(D)**. Bars indicate the standard deviation of the mean (n=5). Different letters on the bars indicate significant differences between treatments based on Tukey’s test (P<0.05).

To evaluate the effects of nonenzymatic antioxidants, we analyzed levels of GSH and proline (**Figures 7A, B**). Compared with the control, the contents of GSH and proline in the leaves of *P.nicotianae*-infected plants decreased by 47.2% and 39.8%, respectively. Compared with the treatment only inoculated with *P.nicotianae*, the contents of GSH and proline in the leaves of *P.nicotianae*-infected plants treated with AMF or BABA increased by 160.1%, 239.7% and 213.9%, 251.8%, respectively, while the contents of GSH and proline in the leaves of *P.nicotianae*-infected plants joint treated with AMF and BABA increased by 361.2%. The results showed that compared with the control, the application of AMF and BABA could increase the accumulation of GSH and proline in leaves. The joint treatment of AMF and BABA had a synergistic effect on the accumulation of GSH in leaves, but compared with the sole AMF or BABA, the joint treatment of AMF and BABA had no significant effect on the accumulation of proline in leaves (**Figures 7A, B**).

We also analyzed the levels of total phenols and flavonoids in tobacco leaves, and the effects of *P.nicotianae* on the contents of GSH and proline were consistent. Compared with the control, the contents of total phenols and flavonoids in tobacco leaves increased by 52.7% and 33.7% after *P.nicotianae* infection. Compared with the sole *P.nicotianae-*infected, the contents of total phenols and flavonoids in leaves of *P.nicotianae*-infected plants treated with AMF or BABA increased by 11.4%, 4.2% and 9.7%, 8.8%, respectively, while the accumulation of total phenols and flavonoids in *P.nicotianae*-infected plants treated with the joint application of AMF and BABA increased by 24.3% and 39.8%, respectively (**Figures 7C, D**). The results showed that compared with *P.nicotianae*-infected plants, AMF or BABA treatment alone increased the accumulation of total phenols, but had no significant effect on the content of flavonoids. The joint treatment of AMF and BABA increased the accumulation of total phenols and flavonoids, indicating that the synergistic treatment of AMF and BABA had an additive effect on the secondary metabolism of *P.nicotianae*-infected plants.

## 4 Discussion

Both BABA and AMF are common in nature. Nonprotein amino acids have been extensively studied in recent years for their ability to induce disease resistance in plants (Cohen et al., 2016). Previous studies have demonstrated that the AMF colonization of plant roots can provide physiological protection and enhance resistance to a wide range of plant pathogens (Miozzi et al., 2019). AMF infection of host plants promotes plant root growth and induces molecular and biochemical responses in plants (Nair et al., 2015). In addition, BABA protects *Arabidopsis* from various virulent pathogens by enhancing pathogen-specific plant resistance mechanisms (Zimmerli et al., 2001). However, the effects of joint AMF and BABA treatment on plant disease resistance, as well as the mechanisms underlying these effects, had not been explored prior to our study.

*P.nicotianae* is a plant pathogen that poses a serious threat to tobacco cultivation, it has induced significant losses to tobacco production globally (Widmer, 2014). In our study, both BABA and AMF enhanced resistance to *P. nicotianae*, and the sole application of BABA *via* spraying induced more resistance to *P. nicotianae* than the sole application of AMF. We also found that BABA positively affected AMF colonization and that the joint application of AMF and BABA enhanced the resistance of tobacco plants to *P. nicotianae* infection.

Photosynthesis is the physiological basis of plant growth and reflects the growth potential and stress resistance of plants. Plant pathogenic fungi hinder the growth and development of plants by inhibiting photosynthesis (Sun et al., 2019). The application of 1 mM BABA has been shown to enhance the drought resistance of broad bean. The application of BABA increased the leaf relative water content, leaf Pn, Tr, and Gs, but decreased the water use efficiency (Abid et al., 2020). The Gs and Ci of perennial sorghum are significantly lower when pants are treated with AMF than in control plants under nuclide stress (Huang et al., 2015). In our study, infection with *P. nicotianae* significantly reduced Pn, Tr, and Gs, indicating that stomatal limitation is one of the reasons why *P. nicotianae* induces decreases in photosynthesis. Regardless of whether tobacco was infected with *P. nicotianae*, BABA and AMF increased leaf gas exchange parameters, which enhanced leaf photosynthesis, and the joint application of BABA and AMF had an additive effect in promoting leaf gas exchange. This may stem from the fact that BABA induces stomatal closure and the expression of downstream stress resistance genes by promoting the accumulation of abscisic acid in tobacco (Xu et al., 2018). In addition, AMF can reduce the damage caused by adverse environments to photosynthetic components such as photosystem II, reduce stomatal limitation, and enhance Pn (Zhao et al., 2021). The joint application of AMF and BABA synergistically increases the resistance of tobacco to TBS, which results in increases in photosynthesis.

Plants have evolved a complex system to resist infection by various pathogens that combines non-enzymatic and enzymatic mechanisms. In this study, we found that compared with the control, the levels of H_2_O_2_ and MDA in plants infected with *P.nicotianae* were significantly increased. After inoculation with AMF or spraying BABA, the MDA level was significantly reduced and the content of H_2_O_2_ did not change significantly. The levels of MDA and H_2_O_2_ were significantly reduced after the joint treatment of AMF and BABA. BABA treatment has been shown to decrease H_2_O_2_ accumulation and the MDA content in rice seedlings (Goswami et al., 2013). AMF reduces the toxicity of heavy metals by enhancing antioxidant defense (SOD activity, CAT activity, and total antioxidant capacity) to reduce the content of H_2_O_2_ and MDA in maize leaves (Zhan et al., 2018). Similarly, plants infected with brown spot and inoculated with AMF have higher POD, SOD, and CAT activities and a lower content of MDA and H_2_O_2_ (Li et al., 2018). Levels of H_2_O_2_ and MDA in leaves were lower in the sole BABA and AMF treatments, which enhanced antioxidant defense to alleviate oxidative stress caused by TBS.

Several studies have shown that BABA and AMF can regulate the expression of defense-related genes involved in plant defense responses. The POD activity of blueberry leaves increases after BABA treatment, which might be related to increases in blueberry leaf spot resistance. Under salt stress, the activities of CAT, POD, SOD, and APX are higher in AMF-colonized plants than in non-colonized plants (Hashem et al., 2018). These results are consistent with the results of this study. The activity of defense enzymes was higher in both BABA-treated plants and AMF-colonized plants, and the effect of the combined application of BABA and AMF was stronger than the effect of applying either one alone, indicating that BABA and AMF may increase the resistance of tobacco plants to *P. nicotianae* by increasing the activity of defense enzymes, as well as up-regulating the expression of antioxidant enzyme genes and TBS resistance genes. This indicates that the joint application of AMF and BABA can alleviate the membrane lipid peroxidation caused by *P. nicotianae* and reduce the content of MDA and H_2_O_2_ by enhancing the activity of antioxidant enzymes, increasing the content of antioxidant substances, and increasing the content of osmotic adjustment substances, which maintains the stability of the cell membrane and improves resistance to TBS (Qun et al., 2007; Tang et al., 2009; Liu et al., 2016).

In the process of AMF infecting plant lines to form mycorrhizal roots, the plant defense system can be regulated by salicylic acid and jasmonic acid signaling pathways, thereby improving the ability of plants to cope with biotic and abiotic stresses. In particular, the jasmonic acid signal transduction pathway plays a very important role in the process of arbuscular mycorrhizal fungi infecting host and forming mycorrhizal (Jung et al., 2012; Cosme et al., 2016). Hause et al. (Hause, 2002) and Tejeda-Sartorius et al. (Tejeda-Sartorius et al., 2010) showed that the expression of a series of precursor synthesis genes of jasmonic acid, especially *AOC* and *Fad*2, was essential in the formation of mycorrhizal structure. The study found that *Funneliformis mosseae* was inoculated with four different genotypes of tomato jasmonic acid signaling pathway. After the mycorrhizal fungi were colonized in the roots, the early blight inoculation and methyl jasmonate treatment were carried out. The results showed that the induction of AMF infection to improve tomato resistance to early blight was closely related to the JA pathway (Lin et al., 2020). Studies have shown that the induced resistance of BABA in potato, tobacco and pepper is achieved through SA pathway (Siegrist et al., 2000; Eschen-Lippold et al., 2010), and the resistance of grapevine is achieved through JA and ET pathways, respectively (Hamiduzzaman et al., 2005; Sós-Hegedűs et al., 2014). In addition, BABA can promote the accumulation of ABA in plant cells under stress to improve the adaptability to adverse environments (Du et al., 2012).

Plants produce a large number of metabolites through secondary metabolic pathways that play a major role in plant defense responses to biotic and abiotic stresses. N, P, and K are important quality-related elements in tobacco, and GSH, proline, flavonoids, and phenolic compounds are major secondary metabolites that accumulate in response to pathogen attack. In this study, the application of BABA and inoculation with AMF, either alone or in combination, increased the levels of N, P, K, GSH, proline, phenolics, and flavonoids, which may have contributed to increases in the resistance of tobacco to TBS and improved tobacco quality. Research shows that the role of potassium in plant disease resistance is basically confirmed. Potassium can reduce the harm of fungi, bacteria and viruses to crops (Liu et al., 2006). Phosphorus is a constituent element of many important compounds (such as nucleic acids, nucleoproteins, phosphoric acid, etc.) in plants. It is involved in the metabolism of carbohydrates, nitrogen and fat in plants. It can also improve the quality of many fruits, vegetables and food crops, and help to enhance the disease resistance of some plants (Dong and Jin, 2010; Yao et al., 2023). As an essential nutrient element for plant growth and development, nitrogen directly affects the formation of plant morphology, plays an important role in plant defense against pathogen infection, and plays an important role in passive disease resistance and active disease resistance. Similarly, nitrogen can affect the growth, virulence and exogenous nitrogen application of pathogens to affect the pathogenicity of pathogens (Hou and Li, 2021).

Inoculation with AMF has been shown to significantly increase N, P, and K levels in *Dioscorea esculenta* and *D. esculenta* yield (Mukhongo et al., 2017). The increase in proline accumulation by AMF and BABA might stem from the increase in proline synthase activity and the reduction in the abundance or activity of catabolic enzymes (Ahmad et al., 2010). Shelled pistachio kernels infected with *Aspergillus flavus* and treated with 7.5 mM BABA also show high phenol and flavonoid accumulation (Aghdam et al., 2020). The growth parameters, photosynthetic pigment levels, and flavonoid levels are higher in AMF-colonized plants than in non-colonized plants (Aseel et al., 2019), and GSH levels remain high in plants treated with BABA, which is essential for maintaining a balanced redox environment to resist oxidative damage induced by drought (Shaw et al., 2016). AMF-inoculated plants show increases in levels of ascorbic acid and GSH (Villani et al., 2021). Since combined treatment of AMF and BABA resulted in a greater accumulation of GSH, proline, flavonoids, and phenolic compounds than their sole treatment, it is highly possible that AMF and BABA additively stimulated secondary metabolism in tobacco plants, leading to enhanced resistance to TBS.

However, there are also some shortcomings in this study. The mass production of AMF in agricultural production practice limits the large-scale application of arbuscular mycorrhizal biotechnology. Therefore, in the future production practice, for different crops, when studying AMF and inducer resistance, it should be targeted to select “AMF-inducer-host plant-pathogen” for in-depth research to determine the effectiveness and efficiency of AMF on the crop disease, and introduce AMF into agricultural production according to local conditions in order to play its maximum role. In addition, research showed that NO participates in the establishment of defense responses to pathogens (Floryszak-Wieczorek et al., 2012), NO accumulation in BABA-treated plants displayed a time-dependent rise, which indicates that NO might be induced by BABA (Li et al., 2020). In this study, BABA may stimulate the accumulation of NO and enhance the resistance to TBS.

## 5 Conclusion

In our study, both AMF inoculation and BABA application enhanced the resistance of tobacco plants to TBS, and the effects of AMF and BABA application were strongest when they were jointly applied. Our findings confirm that AMF and BABA can increase TBS resistance. Based on these results, the increase in the TBS resistance of tobacco plants by AMF and BABA could be attributed to increases in photosynthesis in plants; increases in the activities of the antioxidant enzymes SOD, POD, CAT, and APX in plants; up-regulation of the expression of the antioxidant enzyme genes encoding *SOD*, *POD*, *CAT*, and *APX* and the TBS resistance gene *Ph*; and increased levels of the antioxidants GSH, total phenols, proline, and flavonoids. Additional studies are needed to characterize the regulatory effects of AMF and BABA on biosynthesis in plants *in vivo* and the application of AMF and BABA in crops in actual production systems. Future efforts should be focused on the regulatory mechanism of BABA promoting AMF colonization, the disease resistance pathways and regulatory genes involved in AMF and BABA, and the application of AMF and BABA in practical production. Of course, all these detailed and in-depth studies must first adhere to the corresponding basic research. Because these basic studies have laid a foundation for a deeper understanding of the disease resistance mechanism of AMF and BABA, and also provided a theoretical basis and technical support for the use of inducers and AMF synergistic induction to improve crop disease resistance in production practice.

## Data availability statement

The original contributions presented in the study are included in the article/**Supplementary Material**. Further inquiries can be directed to the corresponding authors.

## Author contributions

SZ, TL, JL and BC conceived and designed the experiments. Provision of test material by SC and YY. SZ, TL, JL and BC analyzed the data and wrote the manuscript. All authors read and approved the final manuscript.
