# Supplementary figures and images for: Mechanisms associated with the synergistic induction of resistance to tobacco black shank in tobacco by arbuscular mycorrhizal fungi and β-aminobutyric acid
Source: Front Plant Sci. 2023 Jun 26;14:1195932. doi: 10.3389/fpls.2023.1195932 (PMC10330952; doi:10.3389/fpls.2023.1195932)

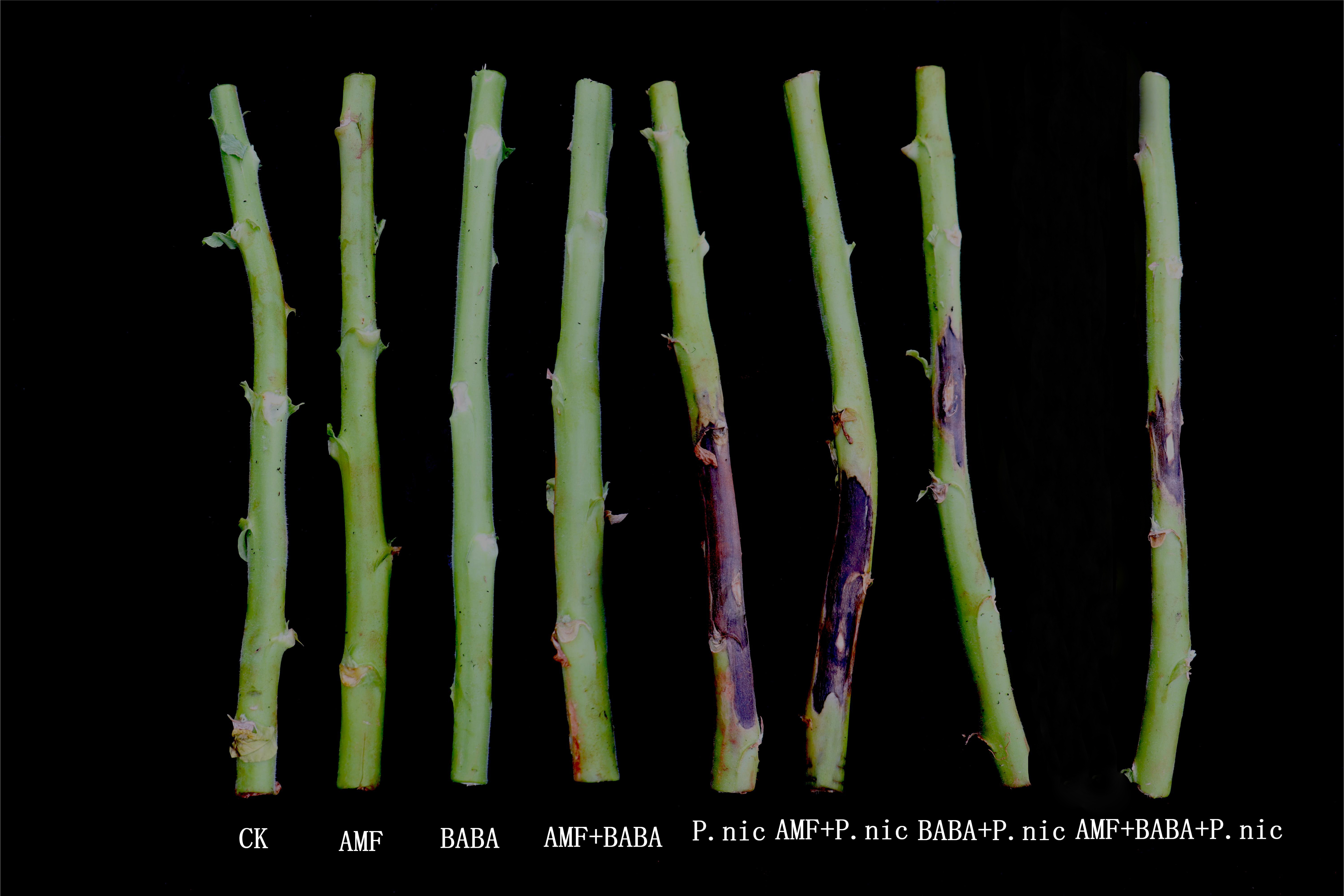

Supplement: Supplementary file 1 [file Image_1.jpeg]
